# Publisher Correction: Enhanced generation and anisotropic Coulomb scattering of hot electrons in an ultra-broadband plasmonic nanopatch metasurface

**DOI:** 10.1038/s41467-017-02106-x

**Published:** 2017-12-12

**Authors:** Matthew E. Sykes, Jon W. Stewart, Gleb M. Akselrod, Xiang-Tian Kong, Zhiming Wang, David J. Gosztola, Alex B. F. Martinson, Daniel Rosenmann, Maiken H. Mikkelsen, Alexander O. Govorov, Gary P. Wiederrecht

**Affiliations:** 1 Center for Nanoscale Materials, Argonne National Laboratory, Argonne, IL 60439 USA; 2 Department of Electrical and Computer Engineering, Duke University, Durham, NC 27708 USA; 3 Institute of Fundamental and Frontier Sciences and State Key Laboratory of Electronic Thin Films and Integrated Devices, University of Electronic Science and Technology of China, Chengdu, 610054 China; 4 Department of Physics and Astronomy, Ohio University, Athens, OH 45701 USA; 5 Materials Science Division, Argonne National Laboratory, Argonne, IL 60439 USA; 6 Department of Physics, Duke University, Durham, NC 27708 USA

*Nature Communications* **8**:986 10.1038/s41467-017-01069-3; Article published online: 17 October 2017

The originally published version of this Article contained an error in Equation 1. The two ℏ terms were missing from this equation, which incorrectly read:

$${\rm{Rat}}{{\rm{e}}_{{\rm{nonthermal}}}} = \mathop {\sum}\nolimits_{i = {\rm{Ag,Au}}} {\frac{{2{e^{\rm{2}}}E_{{\rm{F,}}i}^2}}{{{\pi ^2}{{\left( \omega \right)}^3}}}{\int}_{\!\!\!\!\!{S_{{\rm{NC,}}i}}} {{{\left| {{E_{\rm{n}}}\left( {\theta ,\varphi } \right)} \right|}^2}{\rm{d}}S} } ,$$

The correct form of Equation 1 is as follows:

$${\rm{Rat}}{{\rm{e}}_{{\rm{nonthermal}}}} = \mathop {\sum}\nolimits_{i = {\rm{Ag,Au}}} {\frac{{2{e^{\rm{2}}}E_{{\rm{F,}}i}^2}}{{{\pi ^2}\hbar{{\left(\hbar \omega \right)}^3}}}{\int}_{\!\!\!\!\!{S_{{\rm{NC,}}i}}} {{{\left| {{E_{\rm{n}}}\left( {\theta ,\varphi } \right)} \right|}^2}{\rm{d}}S} } ,$$

This has now been corrected in the PDF and HTML versions of the Article.

