# Supplementary material for: Enhanced generation and anisotropic Coulomb scattering of hot electrons in an ultra-broadband plasmonic nanopatch metasurface
Source: Nat Commun. 2017 Oct 17;8:986. doi: 10.1038/s41467-017-01069-3 (PMC5645345; doi:10.1038/s41467-017-01069-3)
Supplement: Supplementary file 1 — Supplementary Information [file 41467_2017_1069_MOESM1_ESM.pdf]

Supplementary Note 1 | Quantum Theory of Nonthermal Carrier Generation

The classical electrodynamic simulations (Supplementary Figs. 3-7) provide a reliable description of the plasmonic spectra. However, as introduced in the main text, additional efforts are required to reliably predict the generation of high-energy nonthermal carriers. Electrodynamics simulations are based on the bulk dielectric constants of the metals, which become Drude-like in the red and NIR spectral intervals. The Drude model assumes the electron-hole pairs are produced with small excitation energies close to the Fermi level of a metal. In contrast, nonthermal electrons with excitation energies $\sim\hbar\omega$ are created in the Fermi gas due to quantum optical transitions with non-conservation of linear momentum.[1,2] In a large nanostructure, such transitions take place near the surfaces and the hot spots where the linear momentum of a carrier is not conserved due to scattering from the potential wall. As shown in the main text, the rate of optical generation of nonthermal carriers can be calculated according to equation (1).[3] We see that the generation rate includes the factor ω^{-3} arising from the quantum mechanics of optical transitions near a metal surface. In our system, we have two components (Ag nanocube and Au film), and the total generation rate is composed of two terms calculated according to equation (1). In Supplementary Figure 6 we show the calculated rates of nonthermal carrier generation for both Ag and Au components of the metasurface as a function of spacer thickness. As can be seen, the generation rate of excited nonthermal electrons at the gap plasmon resonance is inversely related to the spacer thickness. There are two physical reasons: (1) For small gaps, the hot spots in the system become much stronger and the electric fields increase dramatically. These enhanced electric fields of the gap plasmons are responsible for quantum excitation of hot electrons at the surfaces. (2) The quantum factor ω^{-3} in equation (1) enhances nonthermal electron generation as the plasmon peaks red-shift (Supplementary Fig. 4). This

factor gives rise to the stronger spacer thickness dependence of nonthermal carrier generation than optical absorption (Supplementary Fig. 7c,d).

The distribution of nonthermal carriers generated in the Ag nanocubes or the Au film also appears to be strongly excitation wavelength-dependent, as highlighted in Supplementary Figure 7b. Our calculations indicate nearly 100% of nonthermal carrier generation occurs in the Ag nanocubes when exciting at the multipolar plasmon resonance (at ~370 nm), while 55% and 70% are generated in Au at the gap and quadrupolar plasmon resonances (at ~1000 nm and 630 nm), respectively. Unlike the gap and quadrupolar modes (Fig. 3 and Supplementary Fig. 3c,d,g,h), the multipolar mode is uncoupled from the Au film (Fig. 3 and Supplementary Fig. 3a,e), biasing generation to hot spots at the Ag nanocube corners. We additionally observe a weak linear dependence of the fraction of nonthermal carriers being generated in the nanocubes on the spacer thickness. Due to the higher refractive index of the Al_2O_3 spacer than the PVP/PAH polymer layers, a reduction in spacer thickness decreases the effective index in the gap near the Au surface. As a result, the electric field in the Au film increases more than in the Ag nanocube surface at the gap resonance.

While the above discussion focuses on the generation rate of nonthermal carriers, the decay rate (through e-e scattering) also should impact their contribution to the optical response by modulating the peak nonthermal carrier density (Fig. 4c,d). For a rough estimate of the average nonthermal carrier population during the pump pulse, we can use a simple rate equation to describe the kinetics. Note that the pump pulse duration (~80 fs) is longer than the plasmon dephasing time (estimated from the calculated absorption linewidth as ~ 12 fs), therefore we can use our results from equation (1) of the main text for the CW regime of optical excitation:

$$\frac{dN_{\text{Nonthermal}}}{dt} = Rate_{\text{Nonthermal}} - \frac{N_{\text{Nonthermal}}}{\overline{\tau}_{\text{e-e}}} \tag{1a}$$

$$\overline{\tau}_{\text{e-e}} = \tau_{\text{e-e}}\left(E = E_{\text{F}} + \hbar\omega/2\right) = \tau_0 \left(\frac{2E_{\text{F}}}{\hbar\omega}\right)^2 \tag{1b}$$

Here we estimate the average lifetime for e-e scattering time assuming an energy in the middle of the interval of hot electrons, $E_{\text{F}} < E < E_{\text{F}} + \hbar\omega$. The average number of nonthermal electrons in the nanocube during a square-wave pulse of 80 fs duration is then calculated by:

$$N_{\text{Nonthermal,avg}}\left(t\right) = Rate_{\text{Nonthermal}} \cdot \overline{\tau}_{\text{e-e}}\left(1 - e^{-t/\overline{\tau}_{\text{e-e}}}\right) \tag{2}$$

$$\begin{aligned} N_{\text{Nonthermal,avg}} &= \frac{1}{\Delta t}\int_0^{\Delta t} N_{\text{Nonthermal,avg}}(t)dt \\ &= Rate_{\text{Nonthermal}} \cdot \overline{\tau}_{\text{e-e}}\left(1 - \frac{1 - e^{-t/\overline{\tau}_{\text{e-e}}}}{\Delta t/\overline{\tau}_{\text{e-e}}}\right) \end{aligned} \tag{3}$$

where Δt is the pulse duration. As can be seen from supplementary equation (3), a slower e-e scattering rate should result in a larger buildup of nonthermal carriers during the pulse. For smaller gaps with plasmon resonances in the NIR, this effect should amplify the contribution from nonthermal carriers relative to plasmon resonances in the UV to visible spectrum (Fig. 4d). Supplementary Figure 6 shows the examples of computations for the hybrid Ag-Au plasmonic structures with nano-gaps. This approach was used to compose a global picture (shown as Figure 5 in the main text) for the rates of generation in the hybrid plasmonic systems with and without nano-gaps. Such systems include Au, Ag and Ag-Au nanostructures and the choice of metal and geometry is crucial to obtain efficient hot-carrier production.

To compute the nonthermal populations during the laser pulse in the plasmonic components of our nanostructures (supplementary equations (2) and (3)), we need material

constants of Au and Ag. We can extract these constants from the Drude fits to the empirical dielectric functions.[4] The resulting values for the Drude relaxation rate, plasmon frequency, and Fermi energy can be found in Supplementary Table 1.

Supplementary Note 2 | Nonthermal and Thermal Hot Electron Relaxation

Relaxation of nonthermal carriers is generally treated within the context of Landau's Fermi liquid theory (FLT), whereby the e-e scattering rate is quadratically dependent on the particle energy:

$$\tau_{\text{e-e}}\left(E\right)=\tau_0\frac{E_{\text{F}}^2}{\left(E-E_{\text{F}}\right)^2} \tag{4}$$

$$\tau_0=\frac{128}{\pi^2\sqrt{3}}\omega_{\text{p}}^{-1}=\frac{128}{\pi^2\sqrt{3}}\sqrt{\frac{m^*\varepsilon_0}{ne^2}}\Bigg|_{q\approx 0} \tag{5}$$

where E_{F} is the Fermi energy of the metal, ω_{p} is the intraband (Drude) plasma frequency, m^* is the bulk electron effective mass, n is the electron density, e is the electron charge, and q is the wavevector.[5-7] In the case of nonthermal intraband excitation, electrons and holes are excited up to $\hbar\omega$ from the Fermi energy. Subsequently during e-e scattering, electrons with an energy ΔE relative to the Fermi energy collide with those near the Fermi surface yielding two electrons and a hole with a combined energy of ΔE, conserving both energy and momentum. Thus the nonthermal electron distribution rapidly evolves to a "thermalized" state, increasing the temperature of the electron gas in the process.

Multiple time-resolved two-photon photoemission (2-PPE) studies[5,8-12] have previously been conducted on noble and transition metals with ultrafast resolution (~100 fs). For noble

metals in particular, a good agreement with supplementary equation (4) was found for intraband excitations. However, a consistent deviation from FLT was demonstrated when contributions from interband transitions were involved.[11] Recent theoretical calculations[13-16] have qualitatively reproduced these results, indicating that holes residing in the d-bands of Au and Ag have dramatically lower lifetimes and mean free paths. These calculations have also provided the first indication that local band curvature may impact the e-e scattering rate, with a predicted 3-fold variation in intraband scattering rates near the Fermi surface.[14,16]

An essential assumption in supplementary equation (5) is that the metal can be treated as isotropic with a single effective mass and parabolic band structure. For isotropic band structures within the electron gas model, the wavevector dependence of ω_p is neglected, and transitions are assumed to all occur near the Fermi surface where $q \approx 0$ (Drude approximation). In metals the effect of ω_p on the e-e scattering rate cannot be overstated; it describes the dielectric function (ε) and hence the charge screening of the metal by:

$$\varepsilon(\omega) = \varepsilon_\infty - \frac{\omega_p^2}{\omega(\omega + i\gamma)} \tag{6}$$

where γ is the damping factor. Unlike the simple Drude approximation however, nonthermal electron generation imparts a substantial wavevector (momentum shift) due to the quantum breaking of momentum matching conditions:

$$q(\omega) = \sqrt{\frac{2m^*\omega}{\hbar}} \tag{7}$$

Recent first-principles calculations by Kaltenborn *et al.* have shown the wavevector dependence of the plasma frequency can be estimated as:

$$\Delta\omega_{\mathrm{p}} \approx -\beta q^2 \tag{8}$$

with β being a prefactor. By combining supplementary equations (2), (4), and (5) we arrive at a modified FLT expression for intraband e-e scattering including anisotropy of the band structure:

$$\tau_0 \approx \frac{128}{\pi^2\sqrt{3}}\left(\omega_{\mathrm{p},0} - \frac{2\beta m^*\omega}{\hbar}\right)^{-1} \tag{9}$$

where β and m^* now correspond to scattering within individual (parabolic) conduction bands.

As stated above, the band structure in Ag and Au exhibits a large variability in effective mass at the Fermi surface crossings near the L, X, and K symmetry points of the Brillouin zone (Fig. 8), particularly at the saddle points near the X and L transitions. From supplementary equation (9) one can see carriers excited through quantum intraband transitions along these three bands should have distinct kinetics (from varying effective mass)[7] and distinct spectral signatures arising from differences in their optical permittivity (supplementary equation (6)).[17,18] Assuming FLT applies not only to the bulk effective lifetimes (e.g. from 2-PPE) but also to e-e scattering of carriers in individual bands, supplementary equation (9) can approximate the relative lifetimes for charges near the L, X, and K points from supplementary equation (5). Comparing to bulk 2-PPE measurements in Ag, studies have found a τ_0 of 0.6 fs which corresponds to a minimum lifetime of 14 fs at an 1100 nm pump wavelength assuming a Fermi energy of 5.5 eV.[5,19] Thus even within the isotropic approximation, our results fall within the range of lifetimes predicted from FLT.

After thermalization of the electron gas, the electrons remain in a state of quasiequilibrium with an effective electron temperature much higher than that of the surrounding lattice ($T_{\mathrm{e}} >> T_{\mathrm{L}}$). At this point in time, nonthermal carriers have a negligible contribution to the overall charge density, and the system as a whole can be described using the classical two-

temperature model (TTM).[19-21] In this framework, the thermal electrons lose energy through electron-phonon (e-ph) scattering, which is much faster than e-e scattering for carriers near the Fermi energy. Neglecting e-ph scattering at early times, the maximum electronic temperature can be approximated according to:

$$T_{\mathrm{e}} = \sqrt{T_0 + \frac{2U}{\gamma}} \tag{10}$$

where T_0 is the ambient temperature (300 K), U is the absorbed energy per unit volume, and γ is the temperature dependence of the electronic heat capacity (65 $\mathrm{Jm^{-3}K^{-2}}$ and 66 $\mathrm{Jm^{-3}K^{-2}}$ for Ag and Au, respectively).[21] Assuming a nanocube density of 2 μm^{-2} (Fig. 3d) and 80% of the energy dissipation in the Au (Supplementary Fig. 7) we calculate an electronic temperature of 585 K and 726 K in the Au film and Ag nanocubes, respectively, at the highest measured fluence of 130 μJ cm^{-2}. At these temperatures the e-ph scattering rate is inversely (and nonlinearly) related to T_{e}, and a direct calculation of the predicted thermal electron lifetime is out of the scope of this work. However, we do observe a decreased e-ph scattering rate with increasing fluence. Additionally, previous studies have found values of ~1 ps, similar to those reported here.[19,20]

Supplementary Note 3 | Lifetime Density Analysis

In fitting complex datasets, often multiple solutions of equations are applicable causing the problem to be ill-posed. The use of least-squares regression analysis can thus over- or under-constrain the problem, depending on the number of variables built into the model. This is especially true for transient absorption data, where multiple processes (species) can be present with time-dependent constituent spectra and amplitudes. In the case of a spectral blueshift of a plasmon resonance (e.g. from electron-electron scattering), the overall decay in amplitude is convoluted with a blueshift in the center wavelength of the difference spectrum. The apparent

rate of (amplitude) decay is then dependent on the probe wavelength used, with wavelengths shorter than the transition yielding an effectively slower response than those to the red. Complicating matters, there are often other overlapping transitions or processes present with different kinetics and spectral signatures. In such cases, the LDA method is an incredibly powerful procedure for separating both kinetic and spectral features from data. The LDA procedure was recently detailed extensively by Slavov *et al.*, thus we only present a brief overview here in the context of our data.[22]

The most basic and common procedure for fitting transient absorption data is the global lifetime analysis (GLA) method.[23] This approach involves fitting the time-dependent spectrum (S) to a sum of exponentials involving the various relaxation processes of the system:

$$S_{\mathrm{fit}}\left(\lambda,t\right)=\sum_{i=1}^{N}A_i\left(\lambda,\tau_i\right)\exp\left(-t/\tau_i\right)\otimes IRF\left(t\right) \tag{11}$$

with A being the wavelength-dependent amplitude of each component, commonly known as the decay-associated spectra (DAS), τ the lifetime, and $\otimes$ the convolution with the instrument response function (IRF). In the case of a Gaussian-shaped IRF (Supplementary Fig. 8) with a center of time c and standard deviation σ, the convolution has the analytical form:

$$s_i\left(t\right)=\frac{\exp\left(-k_i t\right)}{2}\exp\left(k_i\left(c+\frac{k_i\sigma^2}{2}\right)\right)\mathrm{erfc}\left(\frac{c+k_i\sigma^2-t}{\sigma\sqrt{2}}\right) \tag{12}$$

with k being the inverse of the lifetime. The amplitudes for each component are then found by linear fitting the product of the pseudoinverse of the guess matrix and the experimental data (S_{exp}) for a given wavelength:

$$\mathbf{A}=\mathbf{S}^{+}_{\mathrm{guess}}\mathbf{S}_{\mathrm{exp}} \tag{13}$$

where S_{guess} is a matrix of the components $s_i(t)$ for each guess of the constituent lifetimes and the "+" superscript indicates the pseudoinverse. In the GLA procedure, the IRF and the lifetimes of the N components are then iteratively fit using the least squares fitting method, which minimizes the square of the residual norm (supplementary equation (14)).

$$\min\left\|\mathbf{S}_{\text{exp}} - \mathbf{S}_{\text{fit}}\right\|_2^2 \tag{14}$$

There are two important points to note about the GLA procedure (supplementary equation (11)). First, the kinetic and spectral contributions for each component are considered separable. Second, the number of components N is fixed and is an assumption made at the outset of the fitting. The former condition precludes any time-dependent shifts of the DAS, while the latter requires assumptions as to the physical processes occurring in the ill-constrained data. Particularly in the case of a lower signal-to-noise ratio, properly selecting the number of components can be difficult and the "optimal" result may be unphysical.

The LDA method is an extension of the GLA approach employing a semi-infinite and quasi-continuous number of components, which relaxes the intrinsic assumptions of GLA. The amplitude $A(\lambda,\tau)$ then represents the inverse Laplace transform of the time-dependent spectrum $S(\lambda,t)$, and can be thought of as a quasi-continuous DAS. In practice, N is discretized with ~100 values taken over a log-scale (in our case from 0.01 to 100 ps). The solution is then found through the process of Tikhonov regularization (TR), whereby a regularization (smoothing) parameter α is used as a low-pass filter to balance the noise introduced from the quasi-continuous number of components and the residual norm. The amplitudes are computed as in supplementary equation (13), however the pseudoinverse of the guess matrix has been modified:

$$\mathbf{S}^{+}_{\text{guess},\alpha} = \left(\mathbf{S}^{\text{T}}_{\text{guess}}\mathbf{S}_{\text{guess}} + \alpha^2\mathbf{L}^{\text{T}}\mathbf{L}\right)^{-1}\mathbf{S}^{\text{T}}_{\text{guess}} \tag{15}$$

where L is the identity matrix. For a range of α the TR is then minimized:

$$\min\left\{\left\|\mathbf{S}_{\exp}-\mathbf{S}_{\text{fit}}\right\|_2^2+\alpha^2\left\|\mathbf{LA}_\alpha\right\|_2^2\right\} \tag{16}$$

and the smoothing norm $\|LA_\alpha\|_2$ is plotted against the residual norm $\|S_{\exp}-S_{fit}\|_2$. The optimal solution is found at the corner of this plot, known as the L-curve, where the experimental data is sufficiently reproduced with low residual while avoiding over-fitting. The corner is defined by the point of maximum curvature (κ):

$$\kappa(\alpha)=\frac{\rho'\eta''-\rho''\eta'}{\left((\rho')^2+(\eta')^2\right)^{3/2}} \tag{17}$$

where ρ and η are the log of the residual and smoothing norms, respectively, and the primes denote differentiation with respect to the regularization factor.[24]

An example of the L-curve analysis is shown in Supplementary Fig. 9. The fitting results for three regularization factors about the corner of the L-curve are shown, with the optimal point highlighted in gold (Supplementary Fig. 9b). An excellent match to the experimental data (Supplementary Fig. 9a) can be seen for all of the fitted spectra (Supplementary Fig. 9c,e,g), confirming all three have similar residuals and goodness of fit. However, as can be seen in their lifetime density maps (LDMs), there are larger and sharper amplitude variations with reduced α (Supplementary Fig. 9d,f,h). Qualitatively, the spectral shapes of the various components remain intact, along with their average lifetimes. However, the components at very short (~0.02 ps) and intermediate (~0.85 ps) lifetimes become washed out with larger smoothing values. Between the points at α = 0.559 and 2.984, there is qualitatively little change observed in the LDM, indicating a stable solution is obtained. The same procedure was performed for all datasets with similar accuracy and stability of solutions.

Finally we consider how the LDA fitting has relaxed the assumptions built into the common GLA method. First, although the number of lifetimes is treated as quasi-continuous, a discrete set of lifetimes appears in the resulting lifetime density maps. As shown in Fig. 7d-g of the main text, these exhibit average lifetimes with a small distribution in each case. The ability to capture the lifetime distribution as opposed to assigning a single ensemble value appears to have particular relevance in systems with energetically or spatially-dependent decay rates, such as nonthermal electrons in metals or emitters coupled to plasmonic nanoantennas, respectively.[14,25] Second, spectral shifts in the data appear as a shearing of the peaks in the LDM versus wavelength, generating ellipticity. This is most apparent for the higher energy transitions such as the multipolar resonance and interband transition in the metasurfaces (Fig. 7a), where blueshifts in the spectra bias the lifetime distributions to larger values at shorter wavelengths. However, this does not change the average or standard deviation of each component's lifetime. In this fashion, both assumptions are circumvented in the implementation of the LDA, and the data is fit with a model-independent method.

Supplementary Note 4 | Separation of Lifetime Distributions and Spectra

From the lifetime density maps (Fig. 7a and Supplementary Fig. 9d,f,h), it is clear that there are a finite number of distinct components with varying spectra at each mode/transition and kinetic distributions that partially overlap at short lifetimes. To disentangle the kinetic and spectral contributions to the LDMs, we applied a global fitting assuming a set of normal distributions:

$$S_{\mathrm{fit}}(\lambda,\tau)=\sum_{i=1}^{N} DAS_i(\lambda)\cdot\left(2\pi\sigma_i^2\right)^{-1/2}\exp\left(-\frac{\left(\tau-\mu_i\right)^2}{2\sigma_i^2}\right) \tag{18}$$

where μ and σ represent to the average and standard deviation of each distribution and N = 6 – 7 to capture the contributions from nonthermal e-e scattering, e-ph scattering, ph-ph scattering into coherent acoustic modes, and semi-infinite components from lattice heating. In each case, the minimum number of distributions was used. Initial locations for each distribution were taken from peaks in the LDM. The generalized global fitting procedure (supplementary equations (10) and (11)) was then employed using the MultiStart algorithm in Matlab to find the optimal solutions. Each wavelength range of the LDM corresponding to the gap, quadrupolar, and multipolar plasmon modes and the interband transition was fitted independently. As can be seen in Fig. 7d-g of the main text, an excellent fit to the LDM data was achieved, and a remarkable consistency in the lifetime distributions was found between all three plasmon resonances (Supplementary Fig. 10).

Supplementary Note 5 | Characteristics of the Nonthermal DAS in Ag

Beginning with the multipolar mode (Fig. 7b), the three nonthermal DAS all have similar spectral lineshapes which blueshift over ~30 nm from the fast to slow carriers, generally indicating a cooling of the carriers. The slow e-e spectrum is centered on that of the e-ph spectrum, both of which match the ground state plasmon resonance wavelength. This indicates that the slow nonthermal carriers represent e-e scattering at energies near the Fermi level.

At the gap mode (Fig. 7c), both fast and intermediate carriers exhibit a strong bleach at the gap resonance, and have zero crossings which are redshifted from the slow nonthermal carriers. As in the case of the multipolar mode, both the slow e-e scattering DAS and e-ph DAS are centered on the resonance wavelength of the gap plasmon in the ensemble. Apart from their near-identical lifetime distributions (Supplementary Fig. 10a-c), this provides further

confirmation that we are probing the same nonthermal carrier populations at all three plasmon modes.

At the gap mode of the 8 nm Al_2O_3 sample, we observe the strongest ultrafast bleach at the peak of the ensemble plasmon resonance (~1020 nm) despite excitation at 1100 nm (Fig. 6b). This is due to the heterogeneous distribution of nanocube sizes (as shown in Supplementary Fig. 2), which leads to excitation of a subset of particles within the ensemble. Since particle sizes with resonances near the resonance of the ensemble are the most prevalent in the film, their contribution to the signal biases the overall response. If there is a homogeneous distribution of nanocubes with a single particle size, one might expect a more symmetric differential absorption signal. In contrast, the asymmetry observed in the nonthermal DAS at the gap resonance (Fig. 7c and Supplementary Fig. 10), as well as the overall transient absorption spectra at short times (Fig. 6a,b), is the result of exciting a finite distribution of particle sizes in the metasurface.

Supplementary Note 6 | Pump Wavelength and Spacer Thickness Dependence

As discussed above, the quantum generation of nonthermal carriers is highly geometry-dependent. To investigate this phenomenon, we performed transient absorption measurements at the gap plasmon resonance on samples with a range of Al_2O_3 spacer thicknesses (Supplementary Figs. 4 and 11). From the DAS for the three e-e components and the e-ph decay, it is possible to approximate the contribution to the hot electron signal at short times arising from the relaxation of the high energy nonthermal carriers:

$$f = \sum_{\lambda} \frac{\left|DAS_{\text{Fast}}(\lambda)\right| + \left|DAS_{\text{Int}}(\lambda)\right|}{\left|DAS_{\text{Fast}}(\lambda)\right| + \left|DAS_{\text{Int}}(\lambda)\right| + \left|DAS_{\text{Slow}}(\lambda) + DAS_{\text{e-ph}}(\lambda)\right|} \tag{19}$$

Here we combine the slow e-e and e-ph spectra as they describe both the growth (through low-energy nonthermal carrier scattering) and decay of the thermalized carrier population, respectively. By taking the magnitude of each DAS and summing over the wavelengths of the gap resonance, we then obtain a measure of the overall signal contribution from the ultrafast nonthermal carriers (Supplementary Fig. 11c). We note this is only an approximation, as the LDA method also includes other terms at time zero arising from longer-lived components (e.g. phonon-phonon scattering), and an exact estimate would require a more targeted analysis method. Nevertheless, we observe a decreased contribution from nonthermal carriers as the spacer thickness increases. We calculate a roughly 30% drop in ultrafast nonthermal signal when increasing the Al_2O_3 thickness from 3 to 25 nm. This is in qualitative agreement with recent kinetic density functional theory predictions[4] for high energy nonthermal carrier generation in hot spots, and is indicative of a surface-mediated effect.[14]

Supplementary Note 7 | Hot Electrons and Coherent Acoustic Modes

Due to the short duration of the excitation pulse relative to the period of phonon modes in the silver nanocubes and gold/oxide films, acoustic phonon modes are excited impulsively in our transient absorption measurements. This gives rise to coherent oscillations of the nanopatch antenna geometry upon lattice thermalization. The deformation of the nanocubes and underlying films modifies the resonance conditions of the gap mode and results in a spectral shift. As can be seen in Supplementary Fig. 12, the metasurface response at long time delays indeed exhibits periodic oscillations about the gap plasmon mode. The initial rise time of the acoustic modes is ~10 ps (Supplementary Fig. 12b), consistent with the phonon-phonon scattering times calculated

in Fig. 7d-g of the main text. The oscillations are then damped over time while heat is lost to the Si substrate and environment, which act as isothermal heat sinks.

We analyzed the metasurface response over a 2 ns window to determine the acoustic phonon modes present. To separate out the exponential and sinusoidal contributions from the data, time traces were first differentiated prior to taking the fast Fourier transform.[26] The result is a spectrogram exhibiting multiple resonances spanning slow (~3 GHz) to fast (~22 GHz) vibrational modes in the system. Previous work has characterized these modes for substrate-coupled silver nanocubes.[27,28] The first mode at ~3 GHz is likely a breathing mode of the gold film, while the higher frequency modes all appear to match acoustic modes of the nanocubes. We have overlaid the dominant asymmetric (A) and symmetric (S) deformation modes as were previously calculated by Petrova *et al.* and observe an excellent agreement with our data. Additional peaks above 15 GHz are also present in our experiments, which are well correlated with higher order asymmetric modes.

We observe coupling to a much larger number of coherent acoustic modes in the nanopatch metasurface than has been demonstrated in previous studies on bare silver nanocubes.[29] The asymmetric excitation of hot electrons at the gap plasmon resonance condition generates a nonuniform initial strain of the lattice during electron-phonon scattering.[30] As the electron pressure exerted on the lattice is proportional to the energy of the electron gas, the large population of highly energetic nonthermal electrons efficiently couples to higher order modes than are typically excited in isolated metallic nanoparticles. For this reason the nanopatch metasurface geometry offers a promising route to populating previously inaccessible acoustic modes for coherent phonon sources and optomechanical transduction.

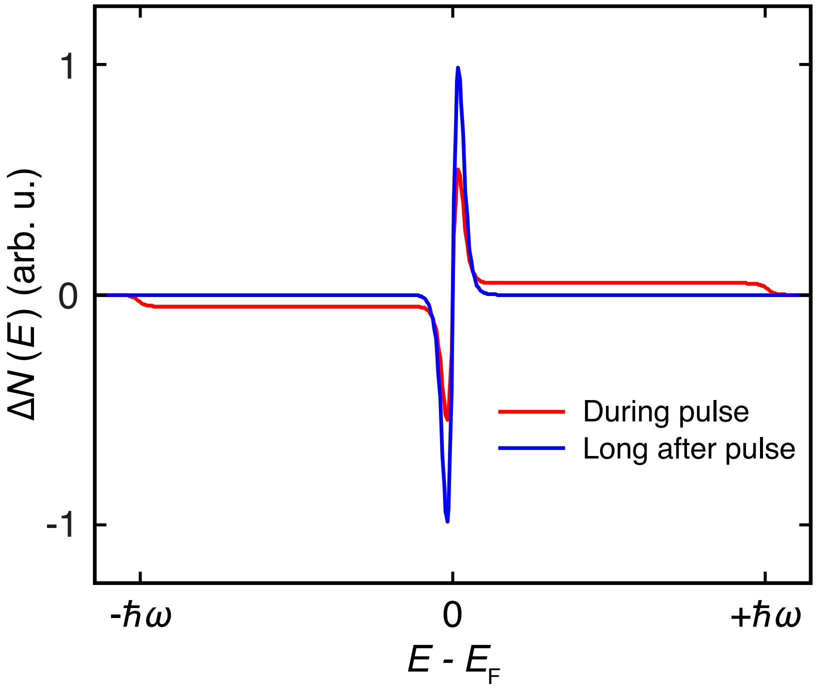

Supplementary Figure 1 | Coexistence of nonthermal and thermal carriers during a finite excitation pulse. In contrast to the idealized diagram of Fig. 1b, which treats all carriers as initially being nonthermal and assumes an instantaneous excitation pulse, in a real system both thermal and nonthermal carriers are generated by an excitation pulse of finite duration. Due to the bulk permittivity of the metal (e.g. Drude response), a fraction of the pulse dissipates through low-energy transitions with negligible momentum change. Relaxation of the nonthermal population through e-e scattering adds to the existing thermal population, until a fully thermalized distribution is achieved. While the nonthermal and thermal populations are indeed distinct types of hot carriers, it is important to note that in reality they evolve both sequentially and in parallel.

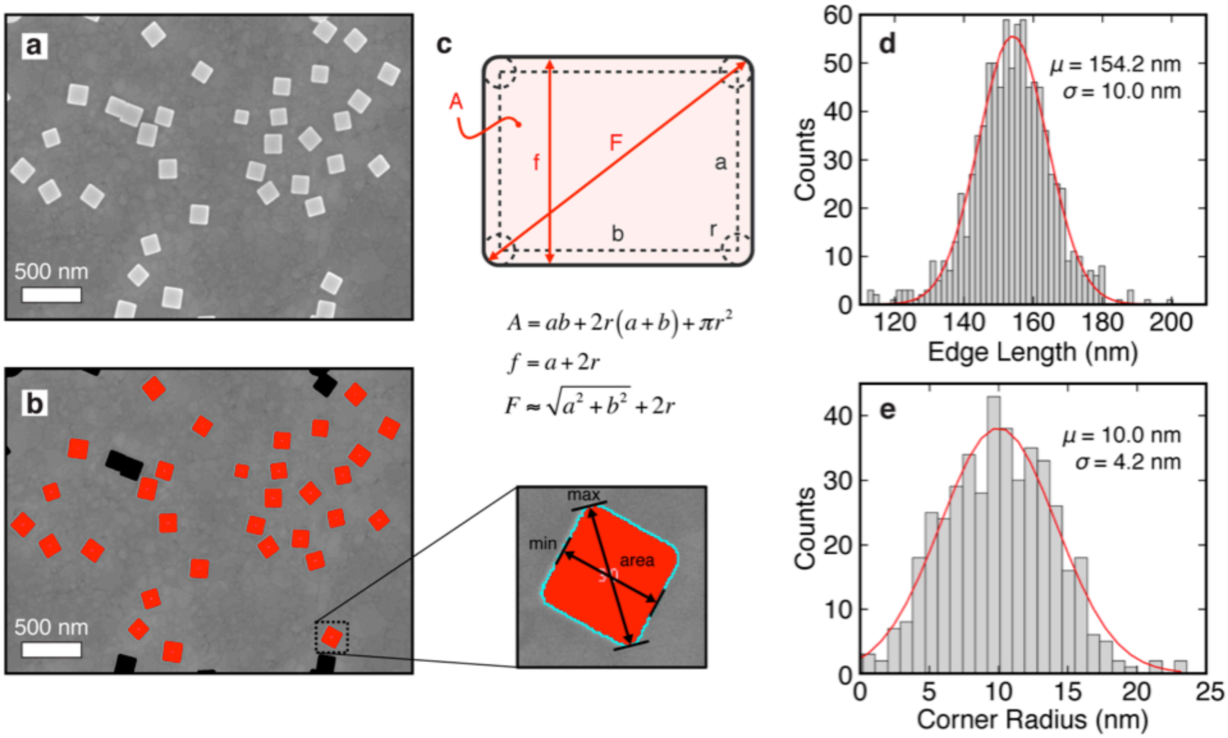

Supplementary Figure 2 | Determination of nanocube dimensions. (**a**) Raw SEM micrograph of silver nanocubes on the metasurface viewed top-down. (**b**) Processed micrograph after particle analysis accounting only for isolated nanocubes. Red regions correspond to areas assigned to each particle, while blue outlines indicate the calculated perimeters. The min/max Feret's diameters and the area were the measured parameters for each nanocube. (**c**) Diagram approximating each nanocube as a rounded rectangle, with measured parameters indicated in red and calculated parameters in black (along with their corresponding relations). (**d**,**e)** Length and corner radius distributions over all measured nanocubes, each fitted to a gaussian distribution to extract their mean and standard deviation (insets).

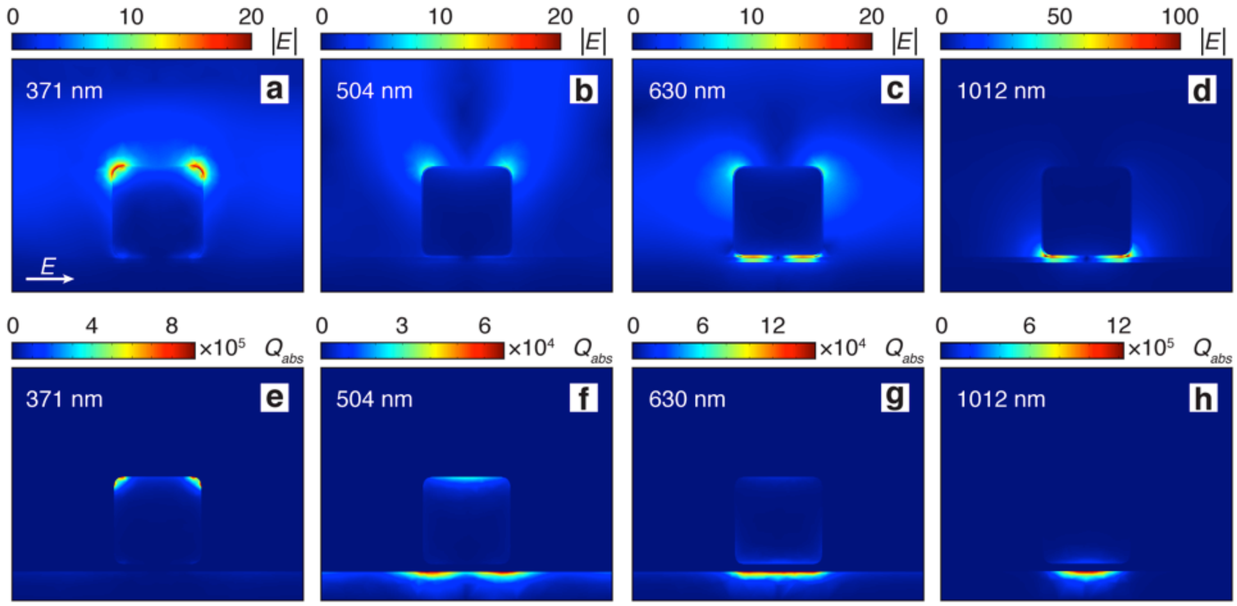

Supplementary Figure 3 | Electric field profiles and power dissipation from classical electrodynamics. (**a-d**) Maps of the electric field magnitude relative to free space for normally incident light polarized in-plane for the 8 nm Al_2O_3 sample at the indicated wavelengths (same as in Fig. 6d). Nonthermal carrier generation scales with the square of the electric field magnitude normal to the metals' surfaces. (**e-h**) Corresponding absorbed power (arbitrary units) through resistive losses calculated using COMSOL. Resistive losses in the metals only account for the direct generation of thermal electrons. They do not predict the hot spot and surface-assisted quantum generation of nonthermal carriers.

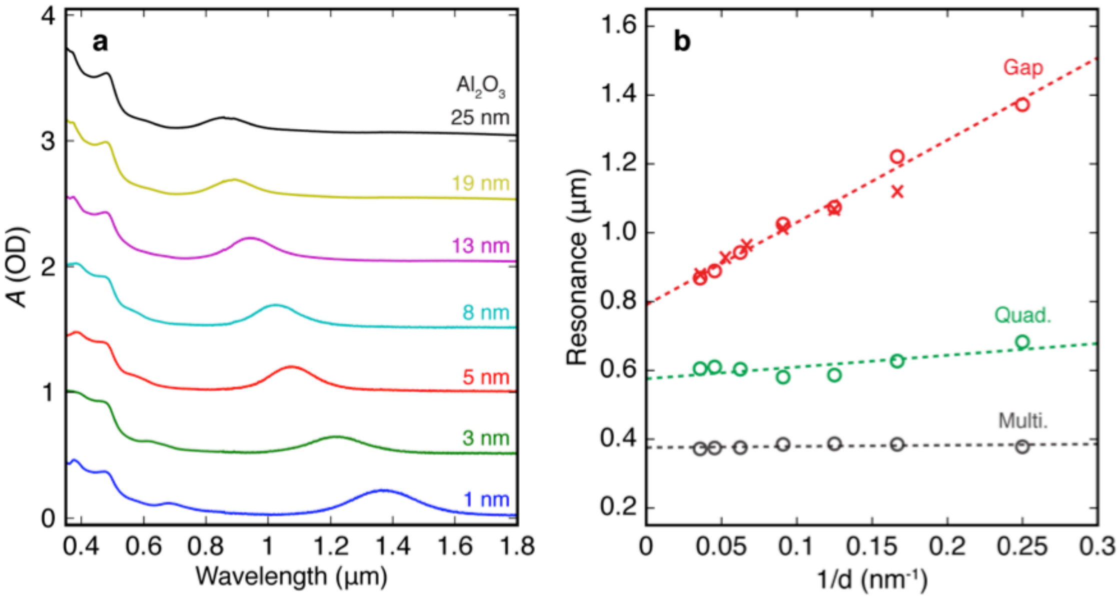

Supplementary Figure 4 | Steady-state absorbance spectra and comparison to simulation. (**a**) Absolute absorbance spectra measured for samples as a function of Al_2O_3 spacer thickness. Individual spectra are offset by 0.5 OD for clarity. Both the quadrupolar and gap plasmon resonances exhibit a blueshift with increased spacer thickness, while the multipolar plasmon resonance and gold interband transition remain fixed. (**b**) Linear dependence of the measured (circles) and simulated (crosses) plasmon resonance wavelengths on the inverse of the total gap thickness (d).

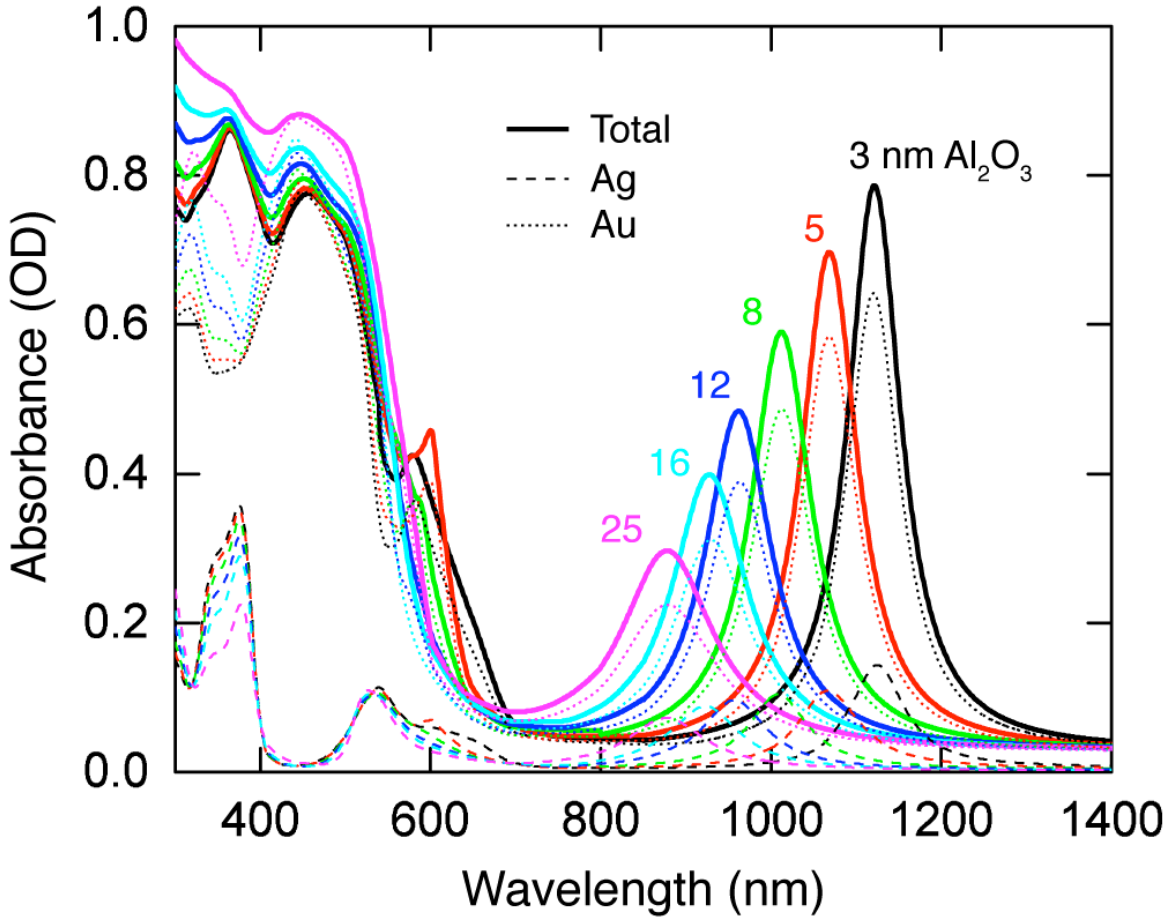

Supplementary Figure 5 | Separated absorption spectra for Ag nanocubes and Au film. Total absorption spectra (solid lines) and the absorption contribution from the Ag nanocubes (dashed lines) and Au film (dotted lines) as a function of Al_2O_3 spacer thickness.

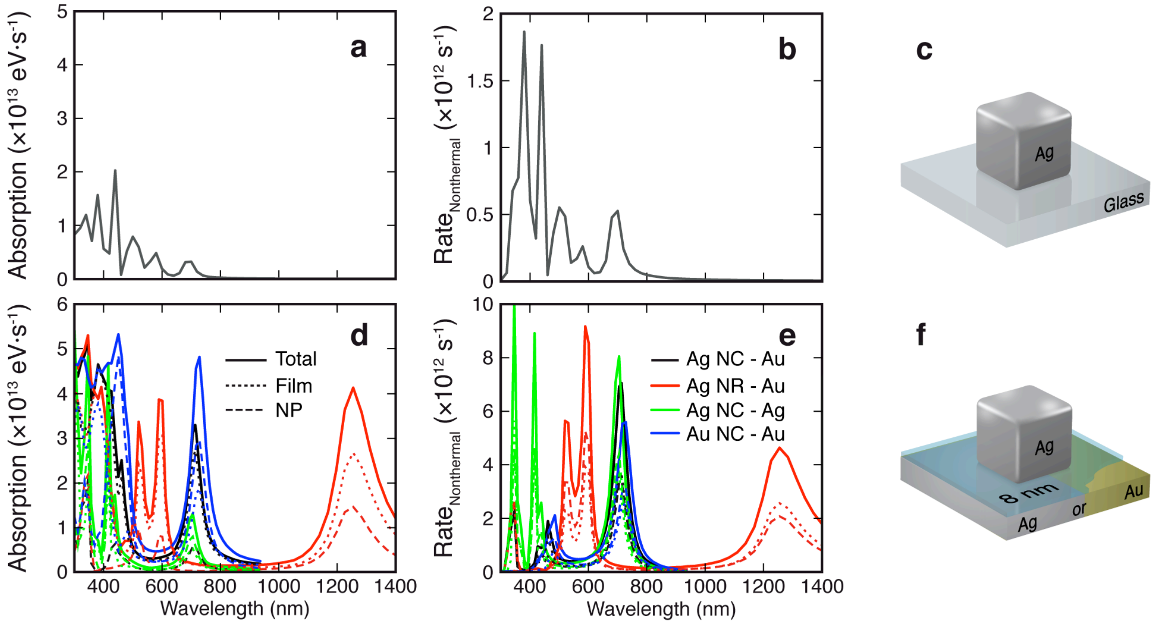

Supplementary Figure 6. Calculations of (a) absorption and (b) nonthermal hot electron generation rates are shown for Ag nanocubes on a glass substrate (c). The enhancement of (d) absorption and (e) nonthermal hot electron generation rates are also shown for Ag nanocubes (NC with dimensions 150 nm x150 nm x150 nm) and nanorods (NR with dimensions of 340 nm x 100 nm x 100 nm) on a Ag or Au substrate with a spacer thickness of 8 nm (f). These data were obtained for relatively small intensity, $I_0 = 3.6 \cdot 10^3$ W/cm^2. For larger intensities, these data can be easily rescaled.

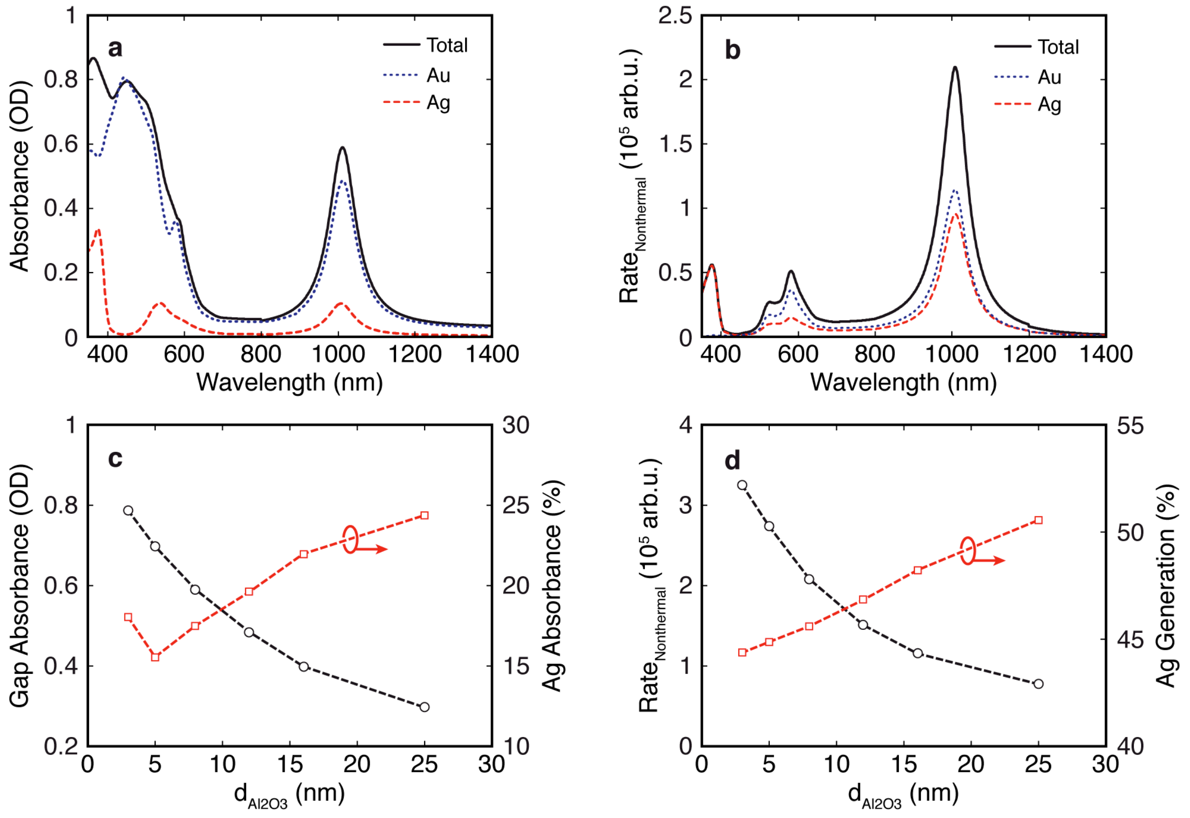

Supplementary Figure 7 | Separated contributions from Ag nanocubes and Au film from simulation. Contributions to steady-state absorbance through resistive losses (**a**) and quantum generation rate of nonthermal carriers (**b**) for the 8 nm Al_2O_3 sample. (**c**) Spacer thickness dependence for total absorbance (black circles) and percent of light absorbed in Ag (red squares) at the gap plasmon resonance. (**d**) Al_2O_3 spacer thickness dependence for the total nonthermal generation rate (black circles) and percent of nonthermal electrons generated in Ag (red squares) at the gap plasmon resonance. Dotted lines in (**c-d**) are guides to the eye.

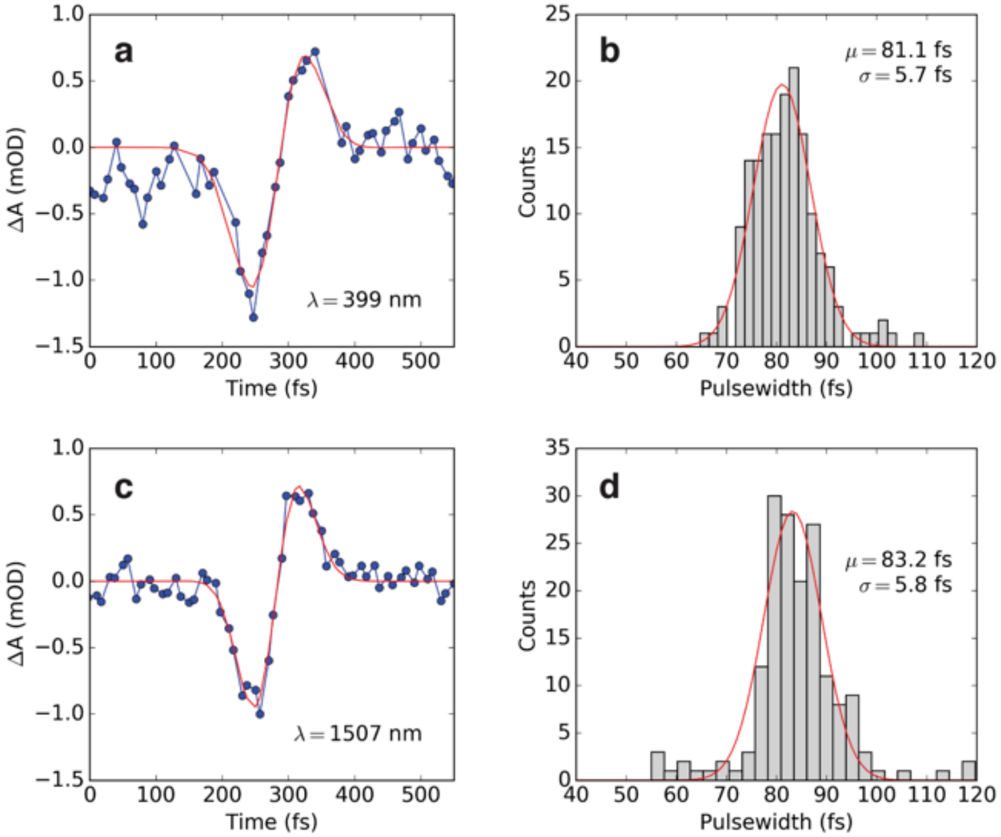

Supplementary Figure 8 | Characterization of instrument response from cross-correlation measurements. (**a**) Optical Kerr response of diamond pumped at 900 nm and probed in the UV-Vis using continuum light from a CaF_2 crystal. The measured data (blue circles) is fitted to a gaussian pulse and its first two derivatives (red line) to extract the cross-correlated pulsewidth of the pump/probe beams. (**b**) Pulsewidth distribution for probe light across the UV-Vis spectrum fitted to a gaussian distribution. (**c**,**d**) Optical Kerr response of diamond pumped at 900 nm and the extracted pulsewidth distribution in the NIR using continuum light generated with a sapphire crystal.

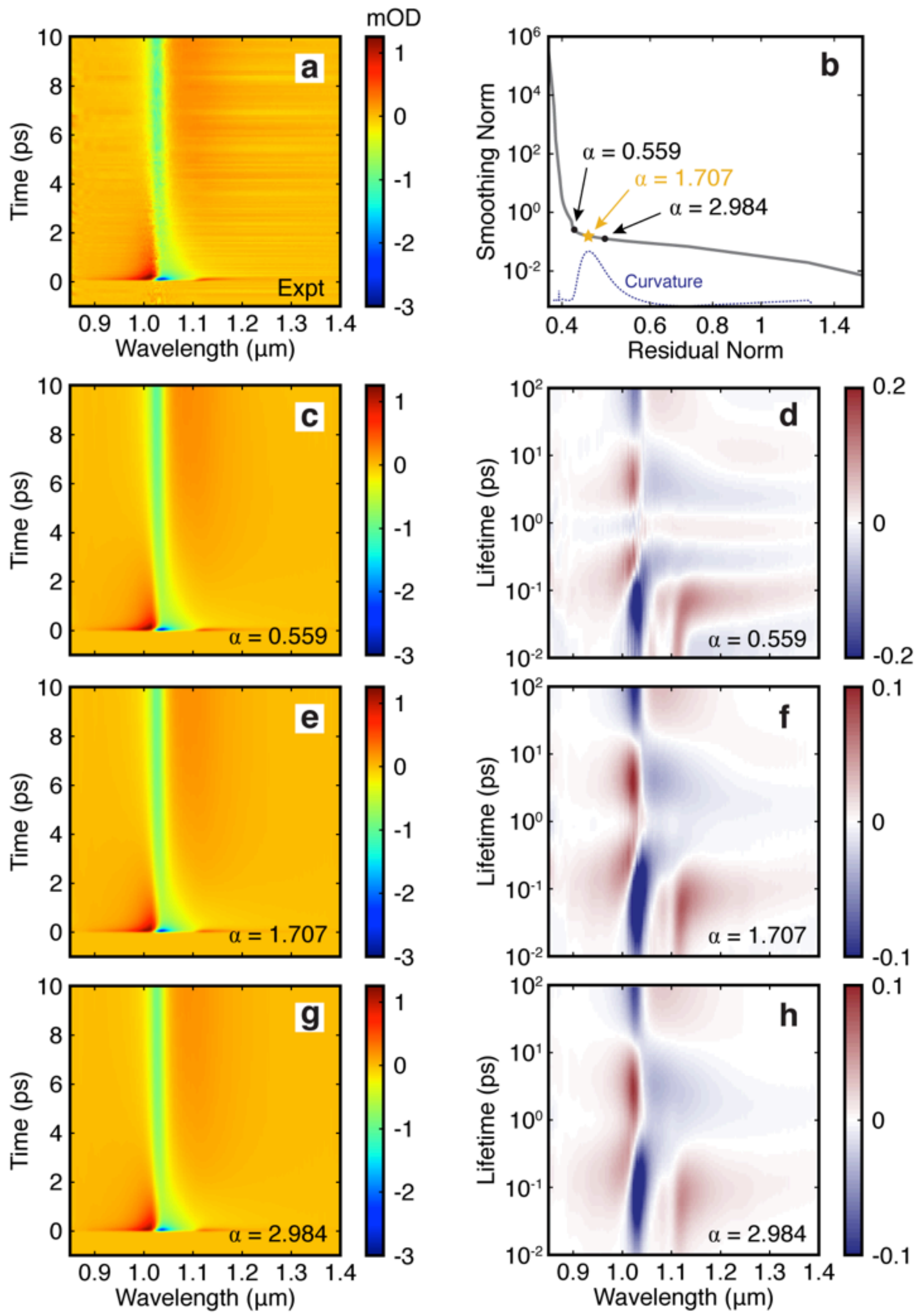

Supplementary Figure 9 | Stability of LDA fitting. (**a**) Differential absorbance spectral map for the 8 nm Al_2O_3 spacer pumped at 1100 nm with a fluence of 20.6 μJ cm^{-2}. (**b**) Corresponding L-curve with three regularization factors selected about the optimal solution (gold), where the curvature (dotted line, linear scaling) is maximized. (**c-h**) Fitted differential absorbance spectral maps (left) and their corresponding lifetime density maps (right) for the three points in (**b**).

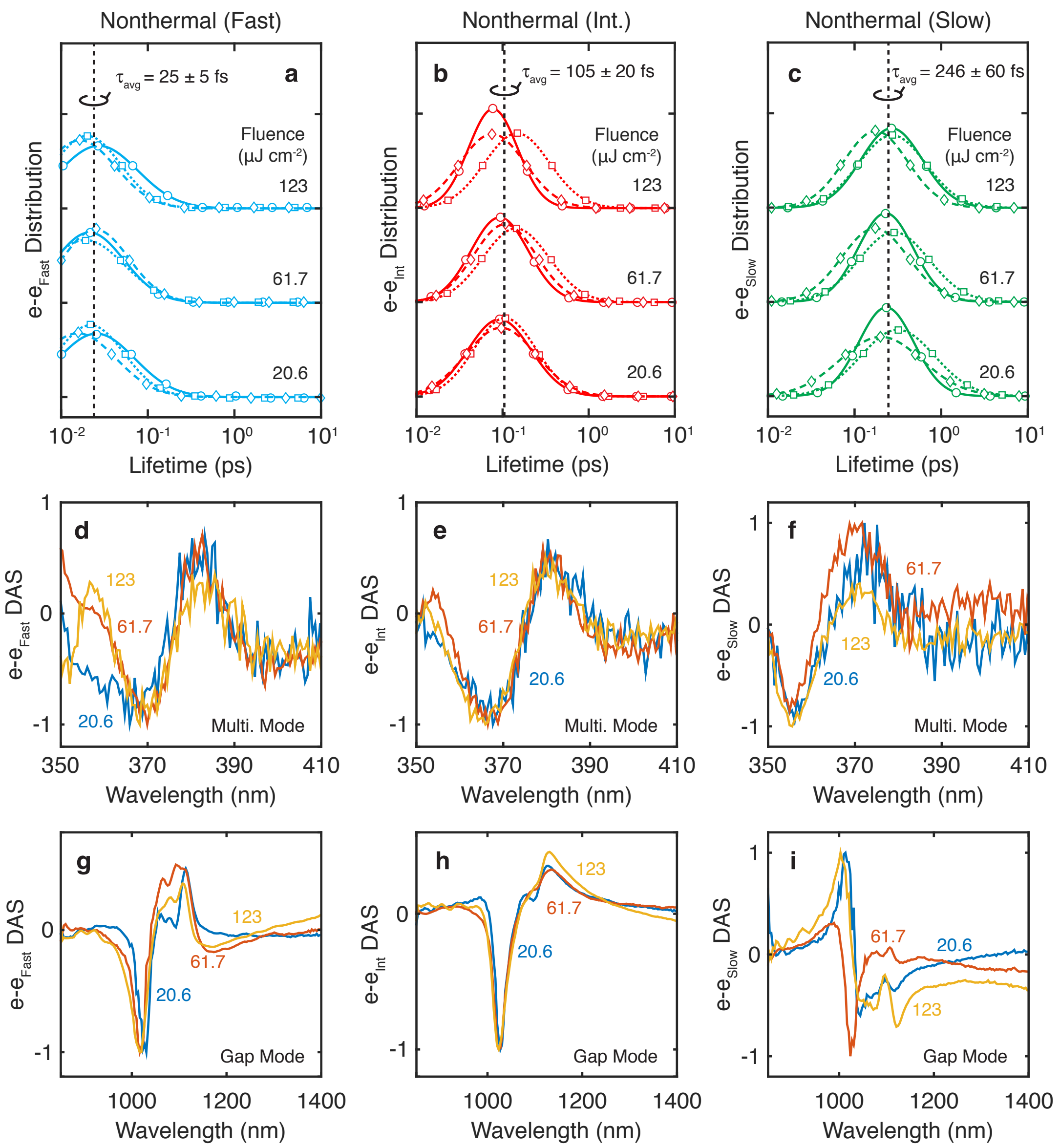

Supplementary Figure 10 | Fluence independence of nonthermal carrier kinetics and spectra at the plasmon modes. (**a-c**) Lifetime distributions of nonthermal e-e scattering at the gap (circles), quadrupolar (squares), and multipolar (diamonds) modes as a function of pump fluence. The average peak lifetimes over all measurements in the 8 nm Al_2O_3 sample are indicated by vertical lines. (**d-f**) Normalized DAS at the multipolar mode and (**g-i**) gap mode for the three nonthermal components as a function of pump fluence. The 61.7 μJ cm^{-2} curve in (i) represents an outlier.

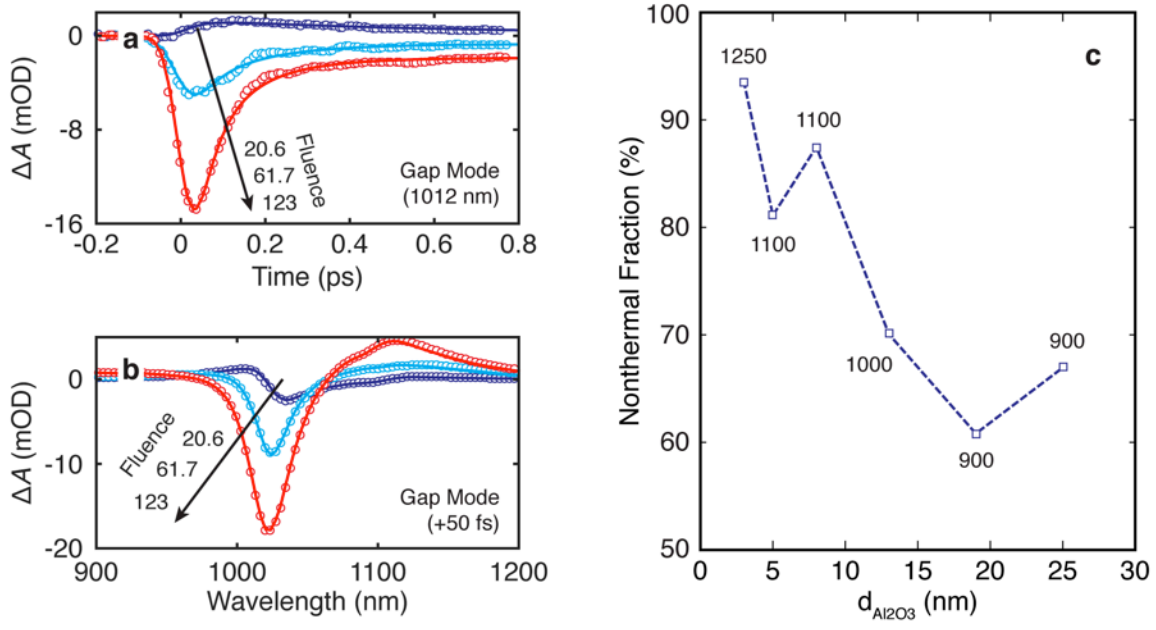

Supplementary Figure 11 | Fluence and spacer thickness dependence of nonthermal contributions. (**a**) Kinetic traces of the measured (circles) and fitted (lines) differential absorbance at short time scales for the 8 nm Al_2O_3 sample as a function of increasing fluence (μJ cm^{-2}). (**b**) Corresponding differential absorbance spectra taken at a delay of 50 fs relative to the pump pulse. An increase can be seen in both the apparent e-e scattering rate along with an inversion of the spectra (blueshift to redshift) with pump fluence, corresponding to a larger signal contribution from fast and intermediate e-e scattering rates. We propose this is the origin of the transition from weak to strong perturbation regime. (**c**) Fraction of hot electron signal arising from fast and intermediate nonthermal carriers as a function of spacer thickness, as calculated using supplementary equation (19). Labels indicate the pump wavelength (in nm) corresponding to each measurement, and pump fluence was kept constant at ~40 μJ cm^{-2}. The dotted line is a guide to the eye.

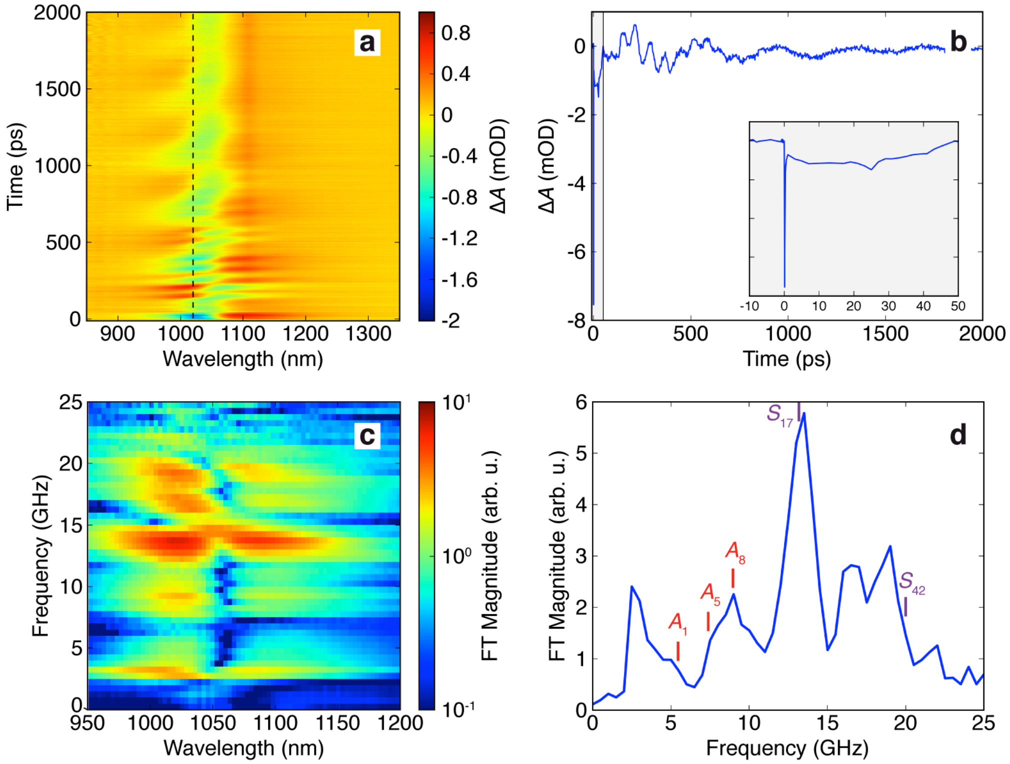

Supplementary Figure 12 | Coherent acoustic phonon modes in the nanopatch metasurface. (**a**) Differential absorbance spectral map for the 8 nm Al_2O_3 spacer at long delay times centered about the gap plasmon mode. Periodic oscillations arise due to expansion/deformation of the nanopatch geometry. Some spectral hole burning at the 1100 nm pump wavelength is observed at long times due to the long duration of scanning. (**b**) Line cut from (**a**) taken at 1020 nm probe wavelength with the initial response (shaded region) expanded in the inset. (**c**) Spectrogram of the data in (**a**) showing the magnitude of the Fourier transform. (**d**) Average magnitude versus frequency for the nanopatch metasurface, with primary eigenvalues of the asymmetric (red) and symmetric (purple) nanocube acoustic modes indicated.[27]

Supplementary Table 1 | Drude parameters fitted to the dielectric functions of Ag and Au. Values are for the damping factor (Drude relaxation rate, γ_p), Drude plasma frequency (ω_p), and Fermi energy (E_F) are all reported in units of eV.

Parameter	Au	Ag
γ_p	0.078	0.02
ω_p	9.1	9.3
E_F	5.5	5.76

Supplementary Table 2 | Fluence dependence of distribution parameters for e-e scattering in the 8 nm Al_2O_3 sample at plasmon resonance. Mean (μ) and standard deviation (σ) of each normal distribution obtained through global fitting. All measurements were performed at a pump wavelength of 1100 nm, and 95% confidence intervals were within 5% of the fitted values.

Fluence ($\mu J\ cm^{-2}$)	Plasmon Res.	μ_{Fast} ($\log_{10}$(ps))	σ_{Fast} ($\log_{10}$(ps))	$\mu_{Int.}$ ($\log_{10}$(ps))	$\sigma_{Int.}$ ($\log_{10}$(ps))	μ_{Slow} ($\log_{10}$(ps))	σ_{Slow} ($\log_{10}$(ps))
20.6	Gap	-1.59	1.04	-1.04	0.862	-0.634	0.750
	Quad.	-1.66	0.922	-0.972	0.847	-0.501	1.00
	Multi.	-1.81	1.04	-1.02	0.968	-0.695	1.12
30.8	Gap	-1.48	1.17	-0.927	0.938	-0.575	0.787
	Quad.	-1.50	1.11	-0.965	0.815	-0.689	0.874
	Multi.	-1.50	1.03	-0.916	0.910	-0.537	1.05
41.1	Gap	-1.52	1.09	-1.06	0.837	-0.420	0.808
	Quad.	-1.52	1.16	-0.917	0.876	-0.520	0.986
	Multi.	-1.67	0.902	-0.980	0.938	-0.636	0.849
61.7	Gap	-1.67	0.943	-1.03	0.785	-0.640	0.750
	Quad.	-1.72	1.06	-0.845	0.902	-0.544	0.954
	Multi.	-1.60	0.905	-0.973	0.854	-0.764	0.906
82.2	Gap	-1.70	0.907	-1.02	0.955	-0.650	0.843
	Quad.	-1.68	1.04	-0.957	0.873	-0.816	0.866
	Multi.	-1.77	0.900	-1.08	0.885	-0.525	1.06
103	Gap	-1.50	1.16	-1.06	0.799	-0.506	0.783
	Quad.	-1.54	1.14	-0.897	0.807	-0.731	0.837
	Multi.	-1.87	1.12	-1.00	0.952	-0.779	0.901
123	Gap	-1.57	1.06	-1.11	0.670	-0.572	0.836
	Quad.	-1.70	0.913	-0.831	0.889	-0.564	0.917
	Multi.	-1.78	0.974	-1.12	0.897	-0.756	0.866

Supplementary Table 3 | Fluence dependence of distribution parameters for e-e scattering in the 8 nm Al_2O_3 sample at gold IB transition. Mean (μ) and standard deviation (σ) of each normal distribution obtained through global fitting. All measurements were performed at a pump wavelength of 1100 nm, and 95% confidence intervals were within 5%, 10%, and 5% of the fitted values for the fast, intermediate, and slow peaks respectively.

Fluence (μJ cm^{-2})	μ Fast (log_{10}(ps))	σ Fast (log_{10}(ps))	μ Int. (log_{10}(ps))	σ Int. (log_{10}(ps))	μ Slow (log_{10}(ps))	σ Slow (log_{10}(ps))
20.6	-1.42	1.14	-0.659	0.846	-0.425	0.795
30.8	-1.33	1.20	-0.709	0.806	-0.518	0.757
41.1	-1.45	1.07	-0.829	0.838	-0.494	0.970
61.7	-1.23	0.989	-0.850	1.04	-0.364	0.580
82.2	-1.25	1.26	-0.828	1.64	-0.524	0.923
103	-1.41	1.06	-0.776	0.820	-0.481	0.855
123	-1.31	1.29	-0.741	0.912	-0.518	0.956

Supplementary Table 4 | Spacer and pump wavelength dependence of distribution parameters for e-e scattering at gap resonance. Mean (μ) and standard deviation (σ) of each normal distribution obtained through global fitting. All measurements were performed at a pump fluence of ~40 μJ cm^{-2} unless otherwise indicated, and 95% confidence intervals were within 5% of the fitted values. *Measurement performed at ~20 μJ cm^{-2}.

d_{Al2O3} (nm)	λ_{pump} (nm)	μ Fast (log_{10}(ps))	σ Fast (log_{10}(ps))	μ Int. (log_{10}(ps))	σ Int. (log_{10}(ps))	μ Slow (log_{10}(ps))	σ Slow (log_{10}(ps))
3	1300*	-1.49	0.963	-1.07	0.754	-0.579	0.741
	1250	-1.71	0.819	-1.22	0.801	-0.600	0.584
5	1200	-1.74	0.901	-1.19	0.769	-0.852	0.715
	1150	-1.38	1.11	-1.09	0.882	-0.697	0.730
	1100	-1.79	0.952	-1.12	0.897	-0.709	0.754
	1050	-1.45	1.10	-1.07	0.744	-0.690	0.632
	1000	-1.56	1.15	-1.09	0.820	-0.931	0.809
8	1100	-1.52	1.09	-1.06	0.837	-0.420	0.808
13	1000	-1.79	0.886	-0.996	1.20	-0.754	0.798
19	900	-1.55	1.11	-1.01	0.889	-0.867	0.825
25	900	-1.59	0.990	-0.963	1.01	-0.739	0.983

Supplementary References

1 Govorov, A. O., Zhang, H. & Gun'ko, Y. K. Theory of photoinjection of hot plasmonic carriers from metal nanostructures into semiconductors and surface molecules. *J. Phys. Chem. C* **117**, 16616-16631 (2013).

2 Govorov, A. O., Zhang, H., Demir, H. V. & Gun'ko, Y. K. Photogeneration of hot plasmonic electrons with metal nanocrystals: Quantum description and potential applications. *Nano Today* **9**, 85-101 (2014).

3 Kong, X.-T., Wang, Z. & Govorov, A. O. Plasmonic nanostars with hot spots for efficient generation of hot electrons under solar illumination. *Adv. Opt. Mater.*, DOI: 10.1002/adom.201600594 (2016).

4 Govorov, A. O. & Zhang, H. Kinetic density functional theory for plasmonic nanostructures: Breaking of the plasmon peak in the quantum regime and generation of hot electrons. *J. Phys. Chem. C* **119**, 6181-6194 (2015).

5 Knoesel, E., Hotzel, A., Hertel, T., Wolf, M. & Ertl, G. Dynamics of photoexcited electrons in metals studied with time-resolved two-photon photoemission. *Surf. Sci.* **368**, 76-81 (1996).

6 Groeneveld, R. H. M., Sprik, R. & Lagendijk, A. Femtosecond spectroscopy of electron-electron and electron-phonon energy relaxation in Ag and Au. *Phys. Rev. B* **51**, 11433-11445 (1995).

7 Kaltenborn, S. & Schneider, H. C. Plasmon dispersions in simple metals and Heusler compounds. *Phys. Rev. B* **88**, 045124 (2013).

8 Bauer, M., Marienfeld, A. & Aeschlimann, M. Hot electron lifetimes in metals probed by time-resolved two-photon photoemission. *Prog. Surf. Sci.* **90**, 319-376 (2015).

9 Fann, W. S., Storz, R., Tom, H. W. K. & Bokor, J. Electron thermalization in gold. *Phys. Rev. B* **46**, 13592-13595 (1992).

10 Petek, H. & Ogawa, S. Femtosecond time-resolved two-photon photoemission studies of electron dynamics in metals. *Prog. Surf. Sci.* **56**, 239-310 (1998).

11 Pawlik, S., Bauer, M. & Aeschlimann, M. Lifetime difference of photoexcited electrons between intraband and interband transitions. *Surf. Sci.* **377**, 206-209 (1997).

12 Ogawa, S., Nagano, H. & Petek, H. Hot-electron dynamics at Cu(100), Cu(110), and Cu(111) surfaces: Comparison of experiment with Fermi-liquid theory. *Phys. Rev. B* **55**, 10869-10877 (1997).

13 Sundararaman, R., Narang, P., Jermyn, A. S., Goddard, W. A., 3rd & Atwater, H. A. Theoretical predictions for hot-carrier generation from surface plasmon decay. *Nat. Commun.* **5**, 5788 (2014).

14 Brown, A. M., Sundararaman, R., Narang, P., Goddard, W. A., 3rd & Atwater, H. A. Nonradiative plasmon decay and hot carrier dynamics: effects of phonons, surfaces, and geometry. *ACS Nano* **10**, 957-966 (2016).

15 Bernardi, M., Mustafa, J., Neaton, J. B. & Louie, S. G. Theory and computation of hot carriers generated by surface plasmon polaritons in noble metals. *Nat. Commun.* **6**, 7044 (2015).

16 Zhukov, V. P., Aryasetiawan, F., Chulkov, E. V., Gurtubay, I. G. d. & Echenique, P. M. Corrected local-density approximation band structures, linear-response dielectric functions, and quasiparticle lifetimes in noble metals. *Phys. Rev. B* **64**, 195122 (2001).
17 Rosei, R. Temperature modulation of the optical transitions involving the Fermi surface in Ag: Theory. *Phys. Rev. B* **10**, 474-483 (1974).
18 Guerrisi, M., Rosei, R. & Winsemius, P. Splitting of the interband absorption edge in Au. *Phys. Rev. B* **12**, 557-563 (1975).
19 Del Fatti, N. *et al.* Nonequilibrium electron dynamics in noble metals. *Phys. Rev. B* **61**, 16956-16966 (2000).
20 Del Fatti, N., Bouffanais, R., Vallée, F. & Flytzanis, C. Nonequilibrium electron interactions in metal films. *Phys. Rev. Lett.* **81**, 922-925 (1998).
21 Voisin, C., Del Fatti, N., Christofilos, D. & Vallée, F. Ultrafast electron dynamics and optical nonlinearities in metal nanoparticles. *J. Phys. Chem. B* **105**, 2264-2280 (2001).
22 Slavov, C., Hartmann, H. & Wachtveitl, J. Implementation and evaluation of data analysis strategies for time-resolved optical spectroscopy. *Anal. Chem.* **87**, 2328-2336 (2015).
23 van Stokkum, I. H., Larsen, D. S. & van Grondelle, R. Global and target analysis of time-resolved spectra. *Biochim. Biophys. Acta* **1657**, 82-104 (2004).
24 Hansen, P. C. & O'Leary, D. P. The use of the L-curve in the regularization of discrete ill-posed problems. *SIAM J. Sci. Comput.* **14**, 1487-1503 (1993).
25 Akselrod, G. M. *et al.* Probing the mechanisms of large Purcell enhancement in plasmonic nanoantennas. *Nat. Photonics* **8**, 835-840 (2014).
26 O'Brien, K. *et al.* Ultrafast acousto-plasmonic control and sensing in complex nanostructures. *Nat. Commun.* **5**, 4042 (2014).
27 Petrova, H. *et al.* Time-resolved spectroscopy of silver nanocubes: Observation and assignment of coherently excited vibrational modes. *J. Chem. Phys.* **126**, 094709 (2007).
28 Szymanski, P., Mahmoud, M. A. & El-Sayed, M. A. The last step in converting the surface plasmonic energy into heat by nanocages and nanocubes on substrates. *Small* **9**, 3934-3938 (2013).
29 Hartland, G. V. Optical studies of dynamics in noble metal nanostructures. *Chem. Rev.* **111**, 3858-3887 (2011).
30 Voisin, C., Del Fatti, N., Christofilos, D. & Vallée, F. Time-resolved investigation of the vibrational dynamics of metal nanoparticles. *Appl. Surf. Sci.* **164**, 131-139 (2000).
